# Sex-specific prognostic utility of the sarcopenia index in all-cause mortality risk for patients with heart failure

**DOI:** 10.3389/fnut.2025.1472596

**Published:** 2025-02-19

**Authors:** Ming Li, Yanying Liang, Baozhen Wu, Ziliang Zhu, Meifang Wang, Jianying Chen, Can Chen

**Affiliations:** ^1^The First Clinical Medical College, Jinan University, Guangzhou, China; ^2^Department of Cardiology, Affiliated Hospital of Guangdong Medical University, Zhanjiang, China

**Keywords:** sarcopenia index, heart failure, all-cause mortality, sex-specific differences, prognostic biomarker

## Abstract

**Background:**

The sarcopenia index (SI), derived from serum creatinine and cystatin C levels, has emerged as a novel and accessible biomarker for predicting clinical outcomes. However, its sex-specific prognostic utility in heart failure (HF) remains poorly understood. This study aimed to investigate the association between SI and all-cause mortality in HF, with a focus on sex-specific differences.

**Methods:**

A retrospective cohort of 753 patients (median age: 69 years; 61% male) diagnosed with HF from a tertiary hospital in China was analyzed. Cox regression models and Kaplan–Meier survival analyses were utilized to evaluate the relationship between SI and all-cause mortality. Stratified analyses based on sex were performed, and the incremental predictive value of SI was assessed by integrating it into traditional risk models.

**Results:**

Over a median follow-up of 537 days, 143 deaths occurred. In adjusted models, a lower SI was significantly associated with an increased risk of all-cause mortality in male patients (hazard ratio: 0.98 per unit increase, 95% confidence interval: 0.97–0.99, *p* = 0.002). Males in the lowest SI tertile had a 1.66-fold higher mortality risk than those in the highest tertile (*p* = 0.004). Kaplan–Meier survival analysis further confirmed these findings, demonstrating significantly lower survival probabilities for males in the lowest SI tertile than for those in higher tertiles (Log-rank *p* = 0.0013). No such association was observed in females. Adding SI to risk models improved prognostic accuracy in males, enhancing the C-statistic from 0.749 to 0.764 and significantly improving net reclassification and discrimination indices (*p* < 0.05).

**Conclusion:**

The SI serves as a robust sex-specific predictor of all-cause mortality in HF, demonstrating significant prognostic value in males but limited utility in females. These findings highlight the potential of SI as a cost-effective addition to existing risk stratification models for male patients with HF.

## Introduction

Heart failure (HF), a complex clinical syndrome marked by escalating global prevalence and healthcare expenditures, poses a formidable challenge to aging societies (1, 2). Beyond its direct hemodynamic consequences, HF frequently intersects with geriatric syndromes that increase morbidity—most notably sarcopenia, the age-related decline in muscle mass and function (3, 4). Compelling evidence links sarcopenia to adverse HF outcomes, including heightened mortality and recurrent hospitalizations, mediated through pathways, including chronic inflammation, insulin resistance, and impaired ventilatory efficiency (5–8). Despite this recognition, clinical translation remains hindered by methodological barriers; the gold-standard sarcopenia assessments via imaging modalities (computed tomography (CT) and magnetic resonance imaging) are cost-prohibitive and logistically impractical for routine risk stratification in HF populations (9, 10).

This gap has sparked interest in biomarker-driven approaches. The sarcopenia index (SI), derived from serum creatinine (Cr)-to-cystatin C ratio (11), has demonstrated acceptable diagnostic accuracy in identifying individuals with sarcopenia [males: area under the curve (AUC) = 0.731; females: AUC = 0.711] (12). Previous research has indicated SI as a cost-effective, easily accessible, and dependable method for predicting muscle health, encompassing aspects—including muscle mass, strength, and function—and for evaluating nutritional risk across various medical conditions (13–16). Additionally, recent studies have demonstrated the prognostic utility of the SI, reporting its ability to stratify future major adverse cardiovascular events risk in individuals with coronary artery disease or HF (17, 18).

Despite its promise, SI’s prognostic utility in HF remains underexplored, particularly regarding potential sex-specific variations. Emerging evidence underscores sex-dimorphic pathways in sarcopenia pathophysiology, spanning metabolic, inflammatory, and hormonal factors (19–21). Such disparities also influence biomarker thresholds, as sex-specific skeletal muscle mass cutoffs are critical for predicting clinical outcomes in chronic diseases (22). Although sarcopenia pathophysiology is increasingly understood, prior studies on the SI in HF have employed uniform sex thresholds (23), neglecting sex-specific variations in creatinine-cystatin C dynamics critical for prognostic accuracy. Therefore, this study examined sex-stratified associations between SI and clinical outcomes in HF to enable precision risk stratification.

## Materials and methods

### Study design and participants

This study was a practical investigation that involved a retrospective analysis of 1,765 patients diagnosed with HF at the Cardiology Department of the Affiliated Hospital of Guangdong Medical University between August 2018 and January 2023.

**Figure 1:**
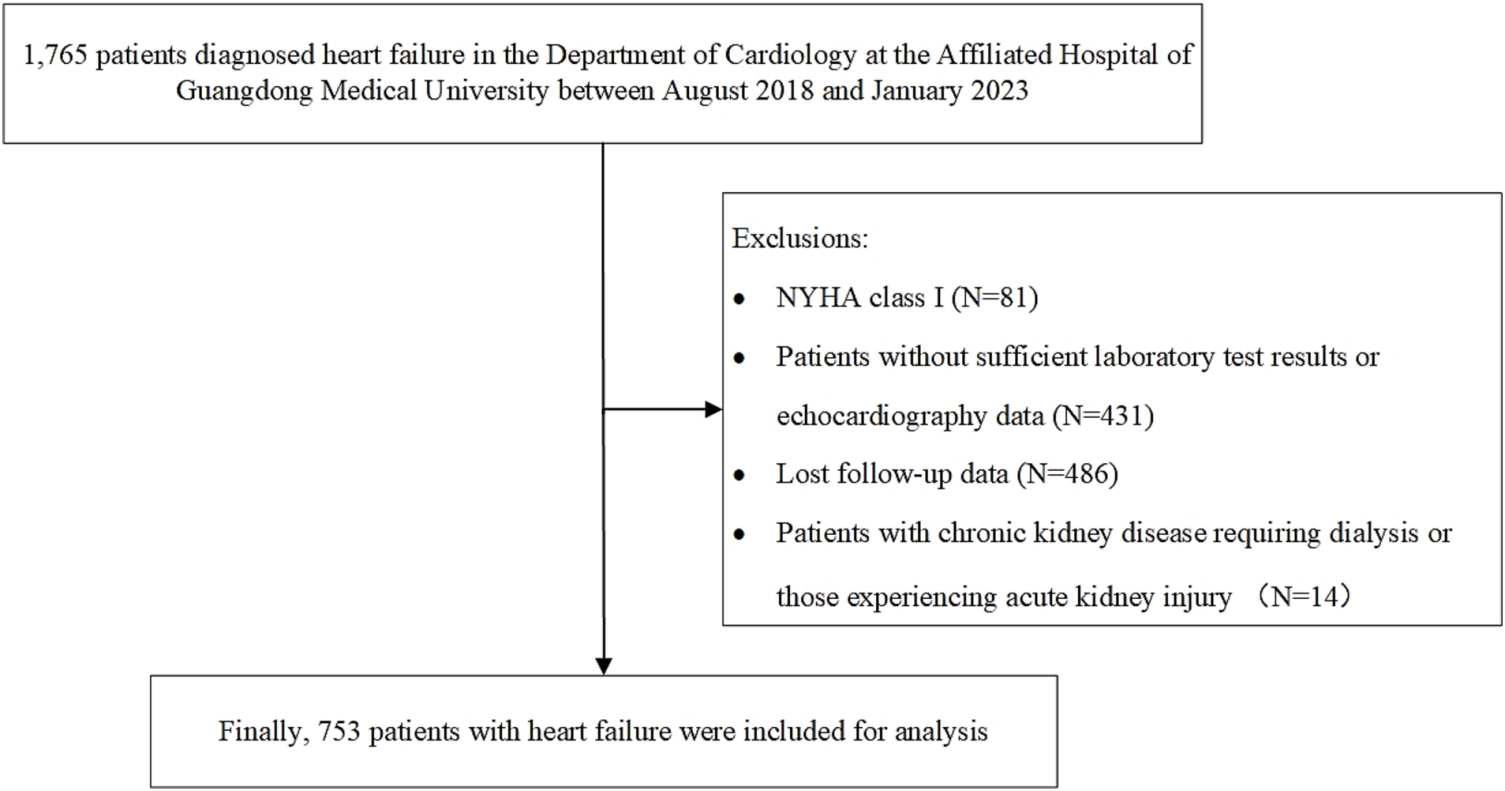
Flow chart for screening patients.

Patients were selected based on the following inclusion criteria: (1) age > 16 years and (2) meeting the diagnostic criteria for HF outlined in the American College of Cardiology/American Heart Association Joint Committee clinical practice guidelines (24). The exclusion criteria were as follows: (1) patients with HF classified as New York Heart Association (NYHA) class I; (2) patients without sufficient echocardiography data or laboratory test results, where the percentage of missing data exceeded 50%; (3) patients with lost follow-up data; and (4) patients with chronic kidney disease requiring dialysis or those experiencing acute kidney injury (16). Finally, 753 patients were included in the analysis (Figure 1).

This study adhered to the principles of the Declaration of Helsinki and was approved by the Ethics Review Committee of the Affiliated Hospital of Guangdong Medical University (Approval Number: PJKT2023-062). Given the retrospective nature of the analysis, the requirement for informed consent was waived (25). Clinical data were extracted from electronic medical records, and follow-ups were conducted via phone calls or electronic medical records.

### Data collection and definitions

Baseline demographic and clinical data [including age; sex; body mass index (BMI); NYHA class; HF etiology; presence of hypertension, atrial fibrillation (AF), or diabetes; laboratory tests; electrocardiogram (ECG) readings; echocardiographic findings; and treatments administered during hospitalization] were collected from the electronic medical recording system. The BMI was determined by dividing weight (Kg) by height squared (m^2^) and categorized as underweight (BMI <18.5 kg/m^2^), ideal (BMI, 18.5–24.9 kg/m^2^), overweight (BMI, 25–29.9 kg/m^2^), or obesity (BMI ≥30 kg/m^2^).

Medical professionals confirmed the etiology of HF based on hospitalization records and categorized it as coronary heart disease, heart valve disease, dilated cardiomyopathy, or hypertrophic cardiomyopathy. AF, hypertension, and diabetes statuses were obtained from self-reports, medical records, or medication use. Various laboratory parameters, including the estimated glomerular filtration rate (eGFR) and the N-terminal pro-B-type natriuretic peptide (NT-proBNP), as well as serum uric acid (SUA), blood albumin (ALB), triglyceride (TG), fasting plasma glucose (FBG), Cr, serum cystatin-C (Cys C), and serum sodium (Na) levels, were analyzed using standard techniques with blood samples collected after overnight fasting (>8 h). NT-proBNP levels were highest during hospitalization.

The triglyceride-glucose (TyG) index was calculated using the following formula: ln(TG [mg/dl] × FBG [mg/dl]/2) (26). The SI was determined using the formula: (Cr [mg/dL]/Cys C [mg/L]) × 100. Hyponatremia was defined as serum Na <135 mmol/L (27), and the eGFR was classified as <60 mL/min/1·73 m^2^ or ≥60 mL/min/1·73 m^2^ (28). ECG data closest to the discharge date were recorded, defining prolonged QTc as a QTc interval > 440 ms (29).

The echocardiographic data followed the guidelines of the American Society of Echocardiography and the European Association of Cardiovascular Imaging, including measurements such as left ventricular end-diastolic diameter, interventricular septal thickness at diastole, left ventricular posterior wall dimensions, left atrial anteroposterior diameter (LA-ap), and pulmonary artery systolic pressure (PASP). The left ventricular mass (LVM) was calculated using the Devereux formula (30, 31). Pulmonary arterial hypertension (PH) was defined as PASP ≥40 mmHg. During hospitalization, medications such as angiotensin-converting enzyme inhibitors (ACEIs)/angiotensin (II) receptor blockers (ARBs)/angiotensin receptor-neprilysin inhibitors (ARNIs), mineralocorticoid receptor antagonists (MRAs), sodium-glucose cotransporter-2 inhibitors (SGLT2is), and *β*-receptor blockers (B blockers) were administered.

### Outcome

The primary endpoint of this study was all-cause mortality, which was assessed by trained medical personnel who contacted the patients or their families by phone. The follow-up time was calculated as the period between the date of HF diagnosis and the date of death or last follow-up. All patients in the study were followed up in November 2023.

### Statistical analysis

For data with less than 20% missing values, the random forest imputation method was employed to handle the missing data. Continuous variables are reported using the means ± standard deviations or medians with interquartile ranges, depending on the distribution being normal or non-normal. Categorical data are presented as numbers and percentages. Group differences in continuous variables were evaluated using either the Student’s *t*-test or Mann–Whitney *U* test, whereas categorical variables were assessed using the Pearson chi-square test or Fisher’s exact test, as appropriate. Cox regression analysis was performed using Firth’s penalized maximum likelihood approach to assess the hazard ratios (HRs) and 95% confidence intervals (CIs) for the association between SI and all-cause mortality.

Four comprehensive models were constructed to account for the potential confounders that could influence all-cause mortality. Model 1 was not adjusted for any variable, providing a crude estimate of the association between SI and mortality. Known variables reflecting disease severity or patient demographics, such as Age, BMI, NYHA class, ejection fraction (EF), and/or sex at baseline, were considered when adjusting for Model 2. Model 3 further incorporated established HF-specific risk factors selected *a priori* based on their prognostic validation in major cohort studies, including PH (32), prolonged QTc (29), NT-proBNP, SUA (33), ALB (34), TyG index (35), hyponatremia (36), and eGFR (28). Model 4 extended Model 3 by including guideline-directed medical therapies for HF (e.g., ACEIs/ARBs/ARNIs, B blockers, MRAs, and SGLT2is) to evaluate whether SI’s prognostic value persists beyond therapeutic modulation.

The non-linear dose–response relationship between the SI and incident all-cause mortality was evaluated using a restricted cubic spline regression model with three knots. The Kaplan–Meier method was used to calculate the cumulative incidence of all-cause mortality according to the three SI tertiles, and differences among groups were assessed using the log-rank test. In the course of the sensitivity analysis, we classified the SI into two distinct groups (High and Low SI) utilizing the median as a dividing point and subsequently conducted subgroup analyses. Additionally, further subgroup analyses were conducted, specifically categorizing individuals based on baseline characteristics such as age (<70 and ≥70 years), BMI (Underweight, Ideal, Overweight, and Obesity), NYHA class (II, III, and IV), PH, prolonged QTc, NT-proBNP level (<3,980 and ≥3,980 pg/mL), SUA level (<417 and ≥417 μmol/L), TyG index (<8.4 and ≥8.4), hyponatremia, and eGFR (<60 and ≥60 mL/min/1.73 m^2^). These additional subgroup analyses aimed to determine whether there was consistency in the prognostic impact of the SI on all-cause mortality across various high-risk factors and to evaluate any potential interactions among these factors.

Variance inflation factors (VIFs) and tolerances were calculated to assess the presence of collinearity. A VIF value below 5 and a tolerance exceeding 0.1 were considered indicators of negligible collinearity. We used the Least Absolute Shrinkage and Selection Operator (LASSO) method to analyze various variables, including sex, age, BMI, LVM, PH, prolonged QTc, NT-proBNP, SUA, TyG index, hyponatremia, eGFR, ACEIs/ARBs/ARNIs, B blockers, MRAs, and SGLT2is. Next, these variables were filtered into the base model. Subsequently, the SI was introduced as a supplementary variable in the base model, and the fit of the models was compared using criteria such as the Akaike Information Criterion (AIC), Bayesian Information Criterion (BIC), and Nagelkerke *R*^2^. Moreover, the Harrell C statistic, Net Reclassification Improvement (NRI), and Integrated Discriminant Improvement (IDI) were used to assess the progressive predictive significance of the SI.

All statistical analyses were performed using R software (version 4.2.2, R Foundation of Statistical Computing, Vienna, Austria) and MSTATA software.^1^ A two-sided *p*-value <0.05 was considered to indicate statistical significance.

## Results

### Baseline characteristics

Initially, the research participants were categorized based on the SI into three groups known as tertiles, with each tertile comprising 251 patients (Q1, SI < 88.2, 251 patients; Q2, SI ≥88.2 and SI <110, 251 patients; and Q3, SI ≥110, 251 patients). The key demographic and clinical variables of the study population are presented in Table 1. The average age of the entire group was 69 years (mean age of 69 ± 14 years), with a majority being male (459 males, 61.0%). The first tertile (Q1) exhibited the lowest mean SI at 74 ± 11. The second tertile (Q2) had an intermediate mean SI of 99 ± 6, and the third tertile (Q3) had the highest mean SI at 134 ± 27. The distribution of sex and age varied significantly among the groups (*p* < 0.001), with Q3 exhibiting a larger percentage of males and a lower average age than Q1 or Q2. Additionally, a significant difference was found in the prevalence of comorbidities such as heart valve disease, dilated cardiomyopathy, hypertension, AF, diabetes, PH, and hyponatremia across the three SI groups. Briefly, the patients classified in Q1 displayed significantly diminished LVM, lower SUA levels, decreased ALB concentrations, reduced TyG index, a higher percentage of eGFR (≥60 mL/min/1.73 m^2^), and increased utilization of MRA compared to those in Q2 and Q3. No significant differences were observed in the distribution of BMI (*p* = 0.056), NYHA class (*p* = 0.089), coronary heart disease (*p* = 0.101), hypertrophic cardiomyopathy (*p* = 0.292), EF (*p* = 0.058), LA-ap (*p* = 0.572), NT-proBNP (*p* = 0.068), prolonged QTc (*p* = 0.388), ACEI or ARB or ARNI prescriptions (*p* = 0.464), B blocker medication (*p* = 0.895), and SGLT2i usage (*p* = 0.868) across the three groups.

**Table 1:** Patient demographics and baseline characteristics.

Characteristic	Overall *N* = 753^1^	Q1, *N* = 251^1^	Q2, *N* = 251^1^	Q3, *N* = 251^1^	*p*-value^1^
SI, Mean ± SD	102 ± 30	74 ± 11	99 ± 6	134 ± 27	<0.001^2^
Sex, *n*(%)					<0.001^3^
Male	459 (61.0%)	93 (37.1%)	161 (64.1%)	205 (81.7%)	
Female	294 (39.0%)	158 (62.9%)	90 (35.9%)	46 (18.3%)	
Age, Mean ± SD	69 ± 14	73 ± 13	69 ± 12	64 ± 15	<0.001^2^
BMI, *n*(%)					0.056^3^
Low weight	100 (13.3%)	43 (17.1%)	35 (13.9%)	22 (8.8%)	
Ideal	404 (53.7%)	140 (55.8%)	124 (49.4%)	140 (55.8%)	
Over weight	164 (21.8%)	44 (17.5%)	62 (24.7%)	58 (23.1%)	
Obesity	85 (11.3%)	24 (9.6%)	30 (12.0%)	31 (12.4%)	
NYHA class, *n*(%)					0.089^3^
NYHA class II	219 (29.1%)	60 (23.9%)	81 (32.3%)	78 (31.1%)	
NYHA class III	380 (50.5%)	128 (51.0%)	128 (51.0%)	124 (49.4%)	
NYHA class IV	154 (20.5%)	63 (25.1%)	42 (16.7%)	49 (19.5%)	
Coronary Heart Disease, *n*(%)	334 (44.4%)	98 (39.0%)	121 (48.2%)	115 (45.8%)	0.101^3^
Heart Valve Disease, *n*(%)	197 (26.2%)	87 (34.7%)	62 (24.7%)	48 (19.1%)	<0.001^3^
Dilated Cardiomyopathy, *n*(%)	77 (10.2%)	20 (8.0%)	20 (8.0%)	37 (14.7%)	0.015^3^
Hypertrophic Cardiomyopathy, *n*(%)	8 (1.1%)	1 (0.4%)	5 (2.0%)	2 (0.8%)	0.292^4^
Hypertension, *n*(%)	339 (45.0%)	95 (37.8%)	121 (48.2%)	123 (49.0%)	0.020^3^
Diabetes, *n*(%)	191 (25.4%)	47 (18.7%)	65 (25.9%)	79 (31.5%)	0.004^3^
AF, *n*(%)	264 (35.1%)	103 (41.0%)	91 (36.3%)	70 (27.9%)	0.008^3^
LA-ap, Median (IQR)	38 (34, 43)	39 (34, 43)	39 (34, 43)	38 (33, 43)	0.572^5^
LVM, Median (IQR)	228 (169, 287)	201 (150, 262)	228 (175, 287)	249 (187, 316)	<0.001^5^
EF, *n*(%)					0.058^3^
HFpEF	317 (42.1%)	117 (46.6%)	112 (44.6%)	88 (35.1%)	
HFmrEF	150 (19.9%)	51 (20.3%)	47 (18.7%)	52 (20.7%)	
HFrEF	286 (38.0%)	83 (33.1%)	92 (36.7%)	111 (44.2%)	
PH, *n*(%)	205 (27.2%)	81 (32.3%)	69 (27.5%)	55 (21.9%)	0.033^3^
Prolonged QTc, *n*(%)	534 (70.9%)	170 (67.7%)	181 (72.1%)	183 (72.9%)	0.388^3^
NT-proBNP, Median (IQR)	3,976 (1,471, 9,170)	4,377 (1,806, 9,754)	3,230 (1,316, 8,789)	4,029 (1,323, 8,632)	0.068^5^
SUA, Mean ± SD	435 ± 150	396 ± 152	433 ± 131	476 ± 155	<0.001^2^
ALB, Mean ± SD	36.6 ± 5.1	35.4 ± 5.4	37.2 ± 4.6	37.1 ± 5.1	<0.001^2^
TyG, Mean ± SD	8.45 ± 0.59	8.34 ± 0.60	8.45 ± 0.55	8.55 ± 0.59	<0.001^2^
Hyponatremia, *n*(%)	78 (10.4%)	36 (14.3%)	19 (7.6%)	23 (9.2%)	0.034^3^
eGFR, *n*(%)					<0.001^3^
≥60	438 (58.2%)	182 (72.5%)	154 (61.4%)	102 (40.6%)	
<60	315 (41.8%)	69 (27.5%)	97 (38.6%)	149 (59.4%)	
ACEI/ARB/ARNI, *n*(%)	563 (74.8%)	182 (72.5%)	194 (77.3%)	187 (74.5%)	0.464^3^
B blocker, *n*(%)	488 (64.8%)	160 (63.7%)	163 (64.9%)	165 (65.7%)	0.895^3^
MRA, *n*(%)	544 (72.2%)	197 (78.5%)	180 (71.7%)	167 (66.5%)	0.011^3^
SGLT2i, *n*(%)	281 (37.3%)	92 (36.7%)	97 (38.6%)	92 (36.7%)	0.868^3^

^1^Mean ± SD; *n* (%); Median (IQR).

^2One-way ANOVA.^

^3Pearson’s Chi-squared test.^

^4Fisher’s exact test.^

^5Kruskal–Wallis rank sum test.^

SI, sarcopenia index; BMI, body mass index; NYHA, New York heart association; AF, atrial fibrillation; EF, ejection fraction; HFpEF, heart failure with preserved ejection fraction; HFrEF, heart failure with reduced ejection fraction; HFmrEF, heart failure with mildly reduced ejection fraction; LVM, left ventricular mass; LA-ap, left atrial antero-posterior diameter; PH, pulmonary arterial hypertension; NT-proBNP, N-terminal pro-B-type natriuretic peptide; SUA, serum uric acid; ALB, blood albumin; TyG, triglyceride-glucose; eGFR, estimated glomerular filtration rate; ACEI, angiotensin-converting enzyme inhibitors; ARB, angiotensin (II) receptor blockers; ARNI, angiotensin receptor-neprilysin inhibitors; B blocker, β-receptor blocker; MRA, mineralocorticoid receptor antagonist; SGLT2i, sodium-glucose cotransporter-2 inhibitors.

### Association between the SI and all-cause mortality

The SI exhibited distinct associations with all-cause mortality across sex-stratified cohorts in progressive multivariable models (Table 2). In the overall population (*N* = 753), a continuous decrease in SI was significantly associated with elevated mortality risk in Model 1 (unadjusted HR: 0.99, 95% CI: 0.99–1.00, *p* = 0.039) and Model 4 (fully adjusted HR: 0.99, 95% CI: 0.98–1.00, *p* = 0.004). The lowest SI tertile exhibited a 1.25-fold greater likelihood of all-cause mortality (HR: 2.25, 95% CI: 1.34–3.76, *p* = 0.002) than the highest SI tertile, with significant trend tests (*p*-trend = 0.002 in Model 4).

**Table 2:** Association between the SI and all-cause mortality in patients with heart failure.

Variables	Model 1		Model 2		Model 3		Model 4	
	HR (95%CI)	*p*-value	HR (95%CI)	*p*-value	HR (95%CI)	*p*-value	HR (95%CI)	*p*-value
Whole population (*N* = 753)								
SI (continuous)	0.99 (0.99, 1.00)	**0.039**	1.00 (0.99, 1.00)	0.549	0.99 (0.98, 1.00)	**0.015**	0.99 (0.98, 1.00)	**0.004**
Q3	1.00 (Reference)		1.00 (Reference)		1.00 (Reference)		1.00 (Reference)	
Q2	0.87 (0.56, 1.35)	0.521	0.81 (0.51, 1.27)	0.358	1.09 (0.68, 1.74)	0.714	1.17 (0.73, 1.87)	0.510
Q1	1.67 (1.13, 2.48)	**0.010**	1.28 (0.82, 2.02)	0.276	2.12 (1.27, 3.52)	**0.004**	2.25 (1.34, 3.76)	**0.002**
*P* for trend		**0.007**				**0.004**		**0.002**
Men (*N* = 459)								
SI (continuous)	0.99 (0.98, 1.00)	**0.002**	0.99 (0.98, 1.00)	**0.034**	0.99 (0.98, 1.00)	**0.006**	0.98 (0.97, 0.99)	**0.002**
Q3	1.00 (Reference)		1.00 (Reference)		1.00 (Reference)		1.00 (Reference)	
Q2	1.11 (0.66, 1.88)	0.685	1.02 (0.60, 1.75)	0.936	1.33 (0.76, 2.32)	0.313	1.38 (0.79, 2.42)	0.262
Q1	2.36 (1.41, 3.96)	**0.001**	1.91 (1.10, 3.30)	**0.021**	2.74 (1.44, 5.23)	**0.002**	2.66 (1.37, 5.15)	**0.004**
*P* for trend		**0.002**		**0.027**		**0.003**		**0.005**
Women (*N* = 294)								
SI (continuous)	1.00 (0.99, 1.02)	0.369	1.01 (1.00, 1.02)	0.085	1.00 (0.99, 1.01)	0.802	1.00 (0.99, 1.01)	0.991
Q3	1.00 (Reference)		1.00 (Reference)		1.00 (Reference)		1.00 (Reference)	
Q2	0.44 (0.19, 0.99)	0.048	0.34 (0.15, 0.79)	0.012	0.52 (0.22, 1.26)	0.149	0.55 (0.23, 1.34)	0.187
Q1	0.95 (0.49, 1.84)	0.880	0.55 (0.27, 1.12)	0.099	0.97 (0.41, 2.28)	0.940	1.17 (0.48, 2.85)	0.727
*P* for trend		0.545		0.395		0.731		0.463

Boldface indicates statistically significant results (*p* < 0.05). HR, Hazard Ratio; CI, Confidence Interval.

Model 1: no covariates were adjusted.

Model 2: adjusted for Age, BMI, NYHA class, EF, and/or Sex.

Model 3: adjusted for Above + Prolonged QTc, PH, NT-proBNP, Hyponatremia, SUA, ALB, eGFR, and TyG.

Model 4: adjusted for Above + ACEI or ARB or ARNI, B blocker, MRA, and SGLT2i.

SI, sarcopenia index; BMI, body mass index; NYHA, New York heart association; EF, ejection fraction; PH, pulmonary arterial hypertension; NT-proBNP, N-terminal pro-B-type natriuretic peptide; SUA, serum uric acid; ALB, blood albumin; TyG, triglyceride-glucose; eGFR, estimated glomerular filtration rate; ACEI, angiotensin-converting enzyme inhibitors; ARB, angiotensin (II) receptor blockers; ARNI, angiotensin receptor-neprilysin inhibitors; B blocker, β-receptor blocker; MRA, mineralocorticoid receptor antagonist; SGLT2i, sodium-glucose cotransporter-2 inhibitors.

Sex-stratified analyses demonstrated marked divergences in the prognostic impact of the SI. In male patients (*N* = 459), a consistent inverse association between SI and mortality risk was observed across all multivariable models. Model 4, when fully adjusted, revealed the most pronounced effects: each unit decrease in continuous SI corresponded to a 2% elevation in mortality risk (HR: 0.98, 95% CI: 0.97–0.99, *p* = 0.002), while individuals in the lowest SI tertile (Q1) exhibited a 1.66-fold increased hazard compared to those in the highest tertile (Q3) (HR: 2.66, 95% CI: 1.37–5.15, *p* = 0.004). In contrast, no significant associations were detected in females (*N* = 294) for continuous SI or tertile comparisons (all *p*-trend > 0.05 in Models 1–4).

**Figure 2:**
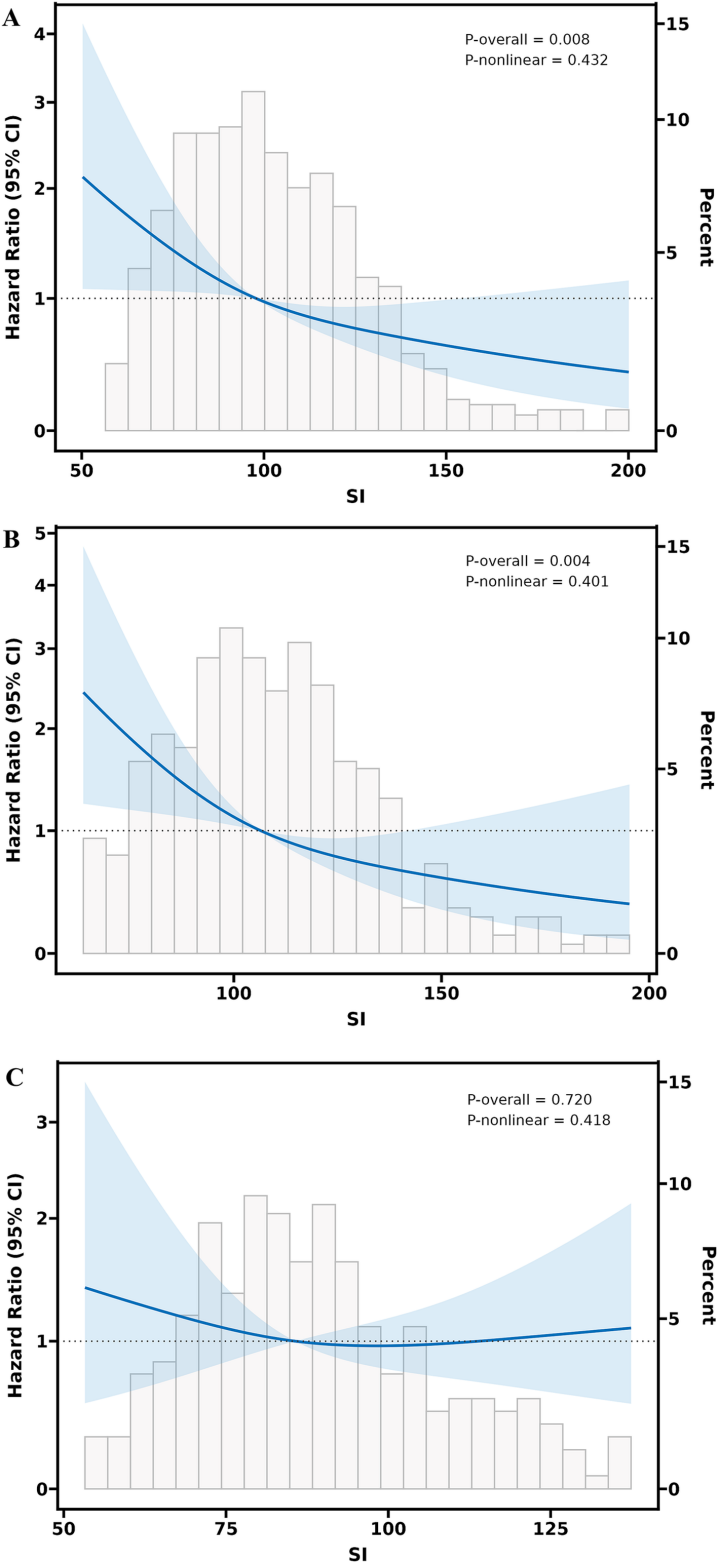
Multivariable adjusted restricted cubic splines regression models between the SI and the risk of all-cause mortality of heart failure. **(A)** Association between the SI and all-cause mortality in the overall population. **(B)** Sex-specific association between the SI and all-cause mortality in male patients with HF. **(C)** Sex-specific association between the SI and all-cause mortality in female patients with HF. The Model with 3 knots located at 10th, 50th, and 90th percentiles. The *Y*-axis represents the HR to present Survival for any value of SI compared to individuals with a reference value (50th percentile) of SI. The Cox regression was adjusted for age, sex, body mass index, New York heart association class, ejection fraction, pulmonary arterial hypertension, prolonged QTC, N-terminal pro-B-type natriuretic peptide, triglyceride-glucose, blood albumin, serum uric acid, estimated glomerular filtration rate, hyponatremia, angiotensin-converting enzyme inhibitors or angiotensin (II) receptor blockers or angiotensin receptor-neprilysin inhibitors, *β*-receptor blockers, mineralocorticoid receptor antagonists, and sodium-glucose cotransporter-2 inhibitors. SI, sarcopenia index; CI, confidence Interval; HF, heart failure.

The SI demonstrated sex-specific prognostic utility across multivariable models (Figure 2). In the overall population, lower SI values exhibited a linear dose–response relationship with increased all-cause mortality risk (*P*-overall = 0.008, *P*-non-linear = 0.432) (Figure 2A). Significant sex differences emerged, with male patients showing a notable inverse association between SI and mortality (*P*-overall = 0.004, *P*-non-linear = 0.401) (Figure 2B), whereas no significant associations were observed in females (*P*-overall = 0.720) (Figure 2C).

**Figure 3:**
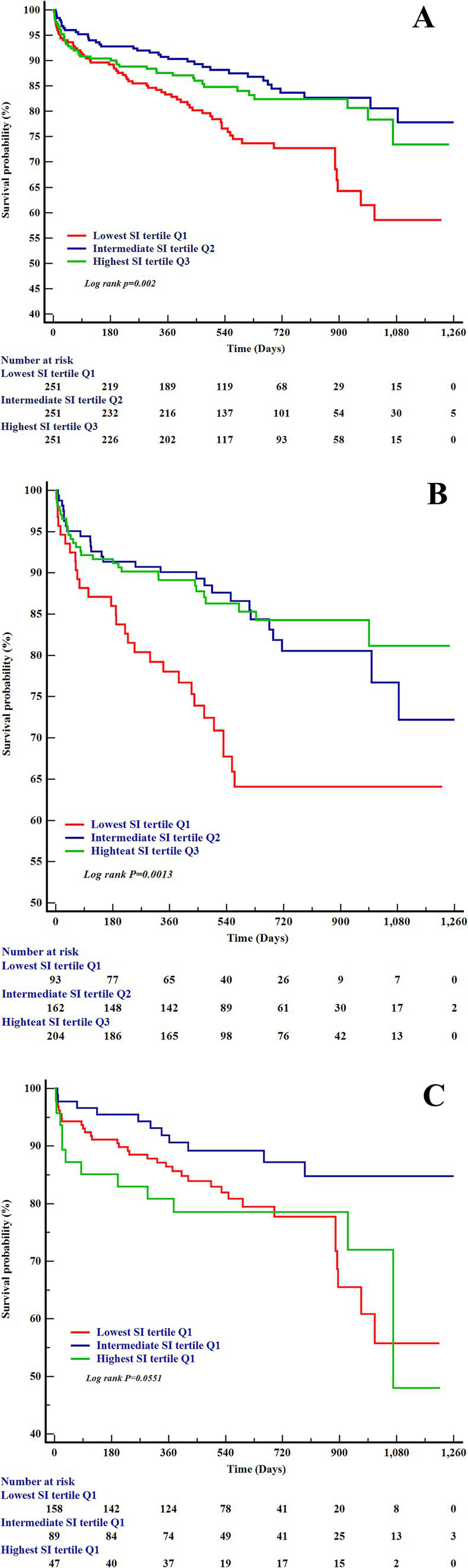
The Kaplan–Meier curves for all-cause heart failure mortality and the SI. **(A)** Kaplan–Meier survival curves by SI tertiles in the overall heart failure cohort. **(B)** Kaplan–Meier survival curves by SI tertiles in male patients with heart failure. **(C)** Kaplan–Meier survival curves by SI tertiles in female patients with heart failure. SI, sarcopenia index.

During a median follow-up of 537 days (IQR: 374–850 days), 143 deaths (18.99% of the cohort) were documented. Kaplan–Meier survival analyses revealed significant disparities in mortality risk across SI tertiles, with pronounced sex-specific patterns (Figure 3). In the overall cohort (Figure 3A), patients in the lowest SI tertile (Q1) exhibited markedly reduced survival probabilities compared to those in the highest tertile (Q3) (Log-rank *p* = 0.002). Sex-stratified analyses further underscored these findings. Males in Q1 exhibited significantly worse survival than those in Q3 (Log-rank *p* = 0.0013; Figure 3B). In contrast, female patients (Figure 3C) showed no statistically significant survival differences across SI tertiles (Log-rank *p* = 0.055).

### Sensitivity and subgroup analysis

**Figure 4:**
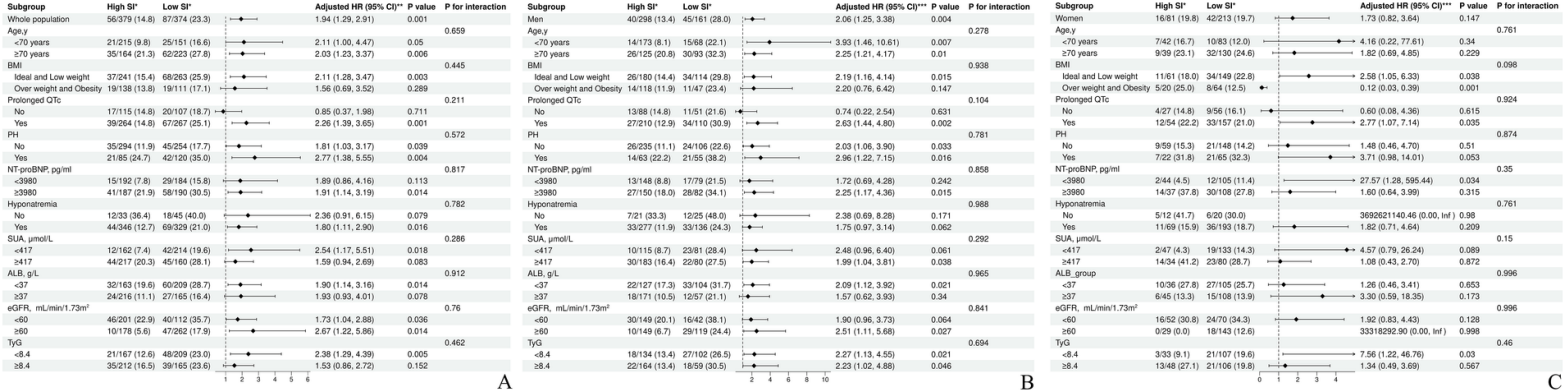
Subgroup analysis of the association between the SI and the high-risk factors of heart failure mortality. **(A)** Subgroup analysis of the association between the SI and the high-risk factors of heart failure mortality in the overall cohort. **(B)** Subgroup analysis of the association between the SI and the high-risk factors of heart failure mortality in male patients. **(C)** Subgroup analysis of the association between the SI and the high-risk factors of heart failure mortality in female patients. *no. of events/total no. (%). **adjusted for sex, age, BMI, NYHA class, EF, Prolonged QTc, PH, NT-proBNP, Hyponatremia, SUA, ALB, eGFR, and TyG, ACEI or ARB or ARNI, B blocker, MRA, SGLT2i. ***adjusted for age, BMI, NYHA class, EF, Prolonged QTc, PH, NT-proBNP, Hyponatremia, SUA, ALB, eGFR, and TyG, ACEI or ARB or ARNI, B blocker, MRA, SGLT2i. SI, sarcopenia index; HR, hazard Ratio; CI, confidence Interval; BMI, body mass index; NYHA, New York heart association; EF, ejection fraction; PH, pulmonary arterial hypertension; NT-proBNP, N-terminal pro-B-type natriuretic peptide; SUA, serum uric acid; ALB, blood albumin; TyG, triglyceride-glucose; eGFR, estimated glomerular filtration rate; ACEI, angiotensin-converting enzyme inhibitors; ARB, angiotensin (II) receptor blockers; ARNI, angiotensin receptor-neprilysin inhibitors; B blocker, β-receptor blocker; MRA, mineralocorticoid receptor antagonist; SGLT2i, sodium-glucose cotransporter-2 inhibitors.

Variables such as age, sex, BMI, NYHA class, EF, PH, prolonged QTc, NT-proBNP, SUA, ALB, TyG index, hyponatremia, eGFR, ACEIs/ARBs/ARNIs, B blockers, MRAs, and SGLT2is were considered when conducting the subgroup analyses. As shown in Figure 4A (overall cohort), Figure 4B (male patients), and Figure 4C (female patients), the SI maintained a consistent association with all-cause mortality across all subgroups. Moreover, no significant interactions were observed between SI and any of the high-risk components of HF mortality (all *p*-values for interaction >0.05).

### Evaluation of the fitting effectiveness and incremental predictive value of the SI

We analyzed the VIF values of the potential variables included in the model. Our results revealed that all VIF values were well below the critical threshold of 5. Moreover, the tolerance of each candidate variable was notably higher than 0.1 (Supplementary Table S1). Using the LASSO regression model, 16 variables (sex, age, BMI, LVM, PH, prolonged QTc, LVM, NT-proBNP, SUA, TyG index, hyponatremia, eGFR, ACEI/ARB/ARNI, B blocker, MRA, and SGLT2i) were identified from the cohort, with nine of these ultimately selected to be included in the basic model (Supplementary Table S2 and Supplementary Figures S1A,B). The fit and predictive power of the SI for all-cause mortality were evaluated using the components of a fitted multivariable Cox regression model.

SI incorporation significantly enhanced model fit, with marked sex-specific disparities (Table 3). In the overall population, the addition of SI to the basic model substantially improved fit, evidenced by lower AIC (1667.5 vs. 1675.5) and AICc (1667.8 vs. 1675.8) values, higher AIC/AICc weights (0.983–0.982 vs. 0.017–0.018), and a marginal increase in Nagelkerke’s *R*^2^ (0.189 vs. 0.177). Sex-stratified analyses revealed striking contrasts. Among males, SI integration markedly improved model performance, with AIC weights rising from 0.005 to 0.995, BIC weights from 0.040 to 0.960, and *R*^2^ increasing from 0.142 to 0.169. In contrast, female models derived no incremental benefit from SI inclusion. Furthermore, SI integration into the prediction model demonstrated incremental prognostic value for all-cause mortality in HF, with pronounced sex-specific heterogeneity (Table 4). In the overall population, the addition of SI significantly improved model discrimination (C-index: 0.756 vs. 0.740) and reclassification performance, evidenced by an NRI of 0.328 (*p* = 0.002) and an IDI of 0.052 (*p* < 0.001). Sex-stratified analyses reinforced these trends. In male patients, SI integration enhanced predictive accuracy (C-index: 0.764 vs. 0.749) and statistically significant NRI/IDI values (both *p* = 0.024). Conversely, it demonstrated negligible contribution to model performance in females, with non-significant net reclassification (NRI = −0.028, *p* = 0.401) and discrimination (IDI = 0.001, *p* = 0.395).

**Table 3:** Performance of the model with the SI in fitting all-cause mortality in HF patients.

	AIC (weights)	AICc (weights)	BIC (weights)	Nagelkerke’s *R*^2^
Whole population				
Basic model	1675.5 (0.017)	1675.8 (0.018)	1717.2 (0.150)	0.177
Basic model + SI	1667.5 (0.983)	1667.8 (0.982)	1713.7 (0.850)	0.189
Men				
Basic model	944.9 (0.005)	945.3 (0.005)	982.1 (0.040)	0.142
Basic model + SI	934.4 (0.995)	934.9 (0.995)	975.7 (0.960)	0.169
Women				
Basic model	549.9 (0.725)	550.5 (0.739)	583.0 (0.943)	0.262
Basic model + SI	551.8 (0.275)	552.6 (0.261)	588.6 (0.057)	0.262

SI, sarcopenia index; AIC, Akaike Information Criterion; BIC, Bayesian Information Criterion.

The basic model included: age, pulmonary arterial hypertension, N-terminal pro-B-type natriuretic peptide, serum uric acid, blood albumin, estimated glomerular filtration rate, hyponatremia, angiotensin-converting enzyme inhibitors or angiotensin (II) receptor blockers or angiotensin receptor-neprilysin inhibitors, and β-receptor blockers.

**Table 4:** Performance of the model with the SI in predicting all-cause mortality in HF patients.

	C-index	Continuous-free NRI		IDI	
	Index	Index	*p-*value	Index	*P*-value
Whole population					
Basic model	0.740 (0.684–0.796)	reference		reference	
Basic model + SI	0.756 (0.702–0.811)	0.328 (0.116–0.431)	0.002	0.052 (0.016–0.100)	0.000
Men					
Basic model	0.749 (0.682–0.816)	reference		reference	
Basic model + SI	0.764 (0.700–0.827)	0.089 (0.035–0.328)	0.024	0.022 (0.002–0.066)	0.024
Women					
Basic model	0.791 (0.722–0.860)	reference		reference	
Basic model + SI	0.796 (0.725–0.866)	−0.028 (−0.120–0.217)	0.401	0.001 (−0.012–0.053)	0.395

SI, sarcopenia index; NRI, net reclassification improvement; IDI, integrated discrimination improvement.

The basic model included: age, pulmonary arterial hypertension, N-terminal pro-B-type natriuretic peptide, serum uric acid, blood albumin, estimated glomerular filtration rate, hyponatremia, angiotensin-converting enzyme inhibitors or angiotensin (II) receptor blockers or angiotensin receptor-neprilysin inhibitors, and β-receptor blockers.

## Discussion

Our investigation revealed a significant inverse association between SI and all-cause mortality in HF, characterized by pronounced sex-specific disparities. In adjusted models, SI exhibited strong prognostic value in male patients, while no comparable association was observed in females. These findings build upon prior research highlighting SI as a readily accessible prognostic marker (18, 37). Additionally, they uniquely underscore biological sex as a critical effect modifier—a previously underexplored dimension in HF biomarker studies.

Emerging evidence underscores the prognostic relevance of the SI across diverse populations. Romeo et al. identified low SI as an independent predictor of long-term mortality and HF readmissions in elderly patients undergoing transcatheter aortic valve replacement (37), while Shi et al. (38) reported an inverse association between SI and cardiovascular morbidity/mortality in the general population. Conversely, Wang et al. (39) demonstrated a non-linear, U-shaped relationship between SI and all-cause mortality in a U.S. cohort. Our findings align broadly with these trends but diverge in demonstrating a linear SI-mortality association in HF, suggesting no safe threshold for SI reduction in this population. These discrepancies in the SI’s prognostic behavior likely reflect variations across study populations. In HF cohorts, the SI appears to operate as a continuous biomarker rather than demonstrating threshold-dependent associations.

Prior investigations have largely utilized unisex analytical frameworks, overlooking potential sex-stratified thresholds for SI interpretation. To the best of our knowledge, this is the first study to investigate the sex-stratified association between SI and all-cause mortality. Our findings demonstrate that SI is inversely associated with long-term all-cause mortality in patients with HF, enhancing model fit and prognostic value. Notably, this association was observed exclusively in males, with no similar correlation in females. The underlying causes of these observed sex differences are likely multifactorial. Differences in muscle strength between males and females may be linked to variations in serum osteocalcin levels (40). Furthermore, the diversity, composition, and metabolic pathways of the gut microbiota may contribute to disparities in muscle mass (41). Vitamin D deficiency significantly increases the risk of sarcopenia in males but not in females (42). Hormone levels, such as testosterone, estrogen, and insulin-like growth factor-1 (IGF-1), also play a role in these differences (43).

Our study results advocate for a sex-stratified approach when assessing muscle mass in patients with HF. When incorporating the simple biological marker SI into HF management, a “one-size-fits-all” biomarker paradigm should be avoided. While the prognostic value of SI is substantial in male patients, its utility appears limited in female patients. Nonetheless, we believe that further multicenter prospective studies may be needed to validate the potential incorporation of SI into risk stratification for prognostic management, particularly for male patients with HF.

### Limitations

This study had some limitations. First, the retrospective, single-center design inherently restricts causal inferences and introduces the potential for selection bias. Unmeasured confounders, including physical activity levels, nutritional status, and treatment adherence, may have influenced both SI values and mortality outcomes. Second, the large number of exclusions may limit the generalizability of our findings, particularly concerning underrepresented subgroups, such as patients with HF who experience improvement in cardiac function to NYHA Class I after treatment. Third, reliance on single-timepoint SI measurements at admission overlooks dynamic fluctuations in muscle mass and renal function during follow-up, potentially misclassifying patients with transient metabolic disturbances. Fourth, the absence of direct skeletal muscle mass quantification using gold-standard modalities, such as DXA, CT, or bioimpedance analysis, precludes definitive diagnostic alignment with established sarcopenia criteria. The exclusive reliance on SI may introduce bias, particularly in patients with advanced renal dysfunction. Finally, this study, which focused on a Chinese population, requires further validation through various ethnic studies.

## Conclusion

Our study identifies SI as a sex-specific predictor of all-cause mortality in HF, with lower SI independently associated with an increased risk in males but not in females. SI incorporation improves risk stratification models in male populations, providing a valuable addition to the conventional management strategies for HF. These findings suggest the potential for a targeted application of SI in male HF risk assessment, pending prospective multicenter validation.

## Data Availability

The raw data supporting the conclusions of this article will be made available by the authors, without undue reservation.

## Footnotes

^1^ https://www.mstata.com
