# Supplementary material for: Sex-specific prognostic utility of the sarcopenia index in all-cause mortality risk for patients with heart failure
Source: Front Nutr. 2025 Feb 19;12:1472596. doi: 10.3389/fnut.2025.1472596 (PMC11879831; doi:10.3389/fnut.2025.1472596)
Supplement: Supplementary file 1 [file Data_Sheet_1.docx]

**Supplementary Table S1. Variance Inflation Factor and Tolerance**

| **Variables** | **VIF (95%CI)** | **SE factor** | **Tolerance (95%CI)** |
| --- | --- | --- | --- |
| age | 1.26 (1.17, 1.39) | 1.12 | 0.79 (0.72, 0.85) |
| BMI | 1.15 (1.08, 1.27) | 1.07 | 0.87 (0.79, 0.93) |
| Prolonged QTc | 1.07 (1.02, 1.22) | 1.03 | 0.94 (0.82, 0.98) |
| LVM | 1.24 (1.15, 1.36) | 1.11 | 0.81 (0.73, 0.87) |
| PH | 1.17 (1.09, 1.29) | 1.08 | 0.86 (0.78, 0.91) |
| NT-proBNP | 1.70 (1.55, 1.88) | 1.30 | 0.59 (0.53, 0.65) |
| Hyponatremia | 1.22 (1.14, 1.35) | 1.11 | 0.82 (0.74, 0.88) |
| SUA | 1.29 (1.20, 1.42) | 1.13 | 0.78 (0.70, 0.84) |
| ALB | 1.24 (1.16, 1.37) | 1.11 | 0.80 (0.73, 0.86) |
| eGFR | 1.37 (1.27, 1.52) | 1.17 | 0.73 (0.66, 0.79) |
| TyG | 1.17 (1.10, 1.30) | 1.08 | 0.85 (0.77, 0.91) |
| ACEI/ARB/ARNI | 1.38 (1.27, 1.52) | 1.17 | 0.73 (0.66, 0.79) |
| B blocker | 1.17 (1.10, 1.30) | 1.08 | 0.85 (0.77, 0.91) |
| MRA | 1.24 (1.16, 1.37) | 1.11 | 0.81 (0.73, 0.86) |
| SGLT2i | 1.17 (1.10, 1.30) | 1.08 | 0.85 (0.77, 0.91) |

VIF, Variance Inflation Factors; CI, confidence Interval; BMI, body mass index; LVM, left ventricular mass; PH, pulmonary arterial hypertension; NT-proBNP, N-terminal pro-B-type natriuretic peptide; SUA, serum uric acid; ALB, blood albumin; TyG, triglyceride-glucose; eGFR, estimated glomerular filtration rate; ACEI, angiotensin-converting enzyme inhibitors; ARB, angiotensin (II) receptor blockers; ARNI, angiotensin receptor-neprilysin inhibitors; B blocker, β-receptor blocker; MRA, mineralocorticoid receptor antagonist; SGLT2i, sodium-glucose cotransporter-2 inhibitors.

**Supplementary Table S2**. **Cox Regression With Variable Reduction (variables Selected by Regularized Regression)**

| **Variables** | **N** | **Event N** | **HR (95% CI)** | | **p-value** |
| --- | --- | --- | --- | --- | --- |
| age | 753 | 143 | 1.02 (1.01, 1.04) | | 0.001 |
| PH |  |  |  |  |  |
| No | 548 | 80 | — |  |  |
| Yes | 205 | 63 | 1.66 (1.17, 2.36) | | 0.004 |
| NT-proBNP | 753 | 143 | 1.00 (1.00, 1.00) | | 0.004 |
| Hyponatremia |  |  |  |  |  |
| ˂ 135 | 78 | 30 | — |  |  |
| ≥ 135 | 675 | 113 | 0.47 (0.30, 0.73) | | <0.001 |
| SUA | 753 | 143 | 1.00 (1.00, 1.00) | | <0.001 |
| ALB | 753 | 143 | 0.96 (0.93, 0.99) | | 0.023 |
| eGFR |  |  |  |  |  |
| ˂ 60 | 315 | 87 | — |  |  |
| ≥ 60 | 438 | 56 | 0.78 (0.53, 1.14) | | 0.202 |
| ACEI/ARB/ARNI |  |  |  |  |  |
| No | 190 | 55 | — |  |  |
| Use | 563 | 88 | 0.70 (0.48, 1.00) | | 0.052 |
| B blocker |  |  |  |  |  |
| No | 265 | 72 | — |  |  |
| Use | 488 | 71 | 0.73 (0.51, 1.03) | | 0.075 |
| HR, Hazard Ratio; CI, Confidence Interval; PH, pulmonary arterial hypertension; NT-proBNP, N-terminal pro-B-type natriuretic peptide; SUA, serum uric acid; ALB, blood albumin; eGFR, estimated glomerular filtration rate; ACEI, angiotensin-converting enzyme inhibitors; ARB, angiotensin (II) receptor blockers; ARNI, angiotensin receptor-neprilysin inhibitors; B blocker, β-receptor blocker; | | | | | |

**Supplementary Figure S1**. **Variables selection using the LASSO binary logistic regression model**


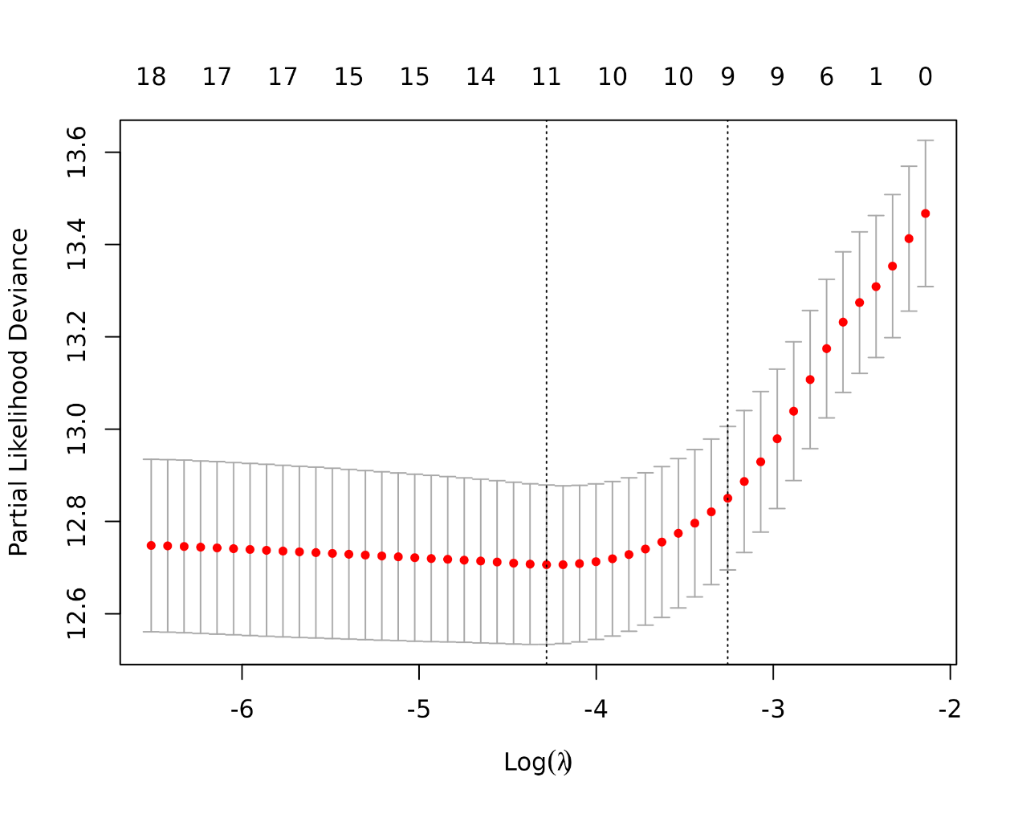


**A**


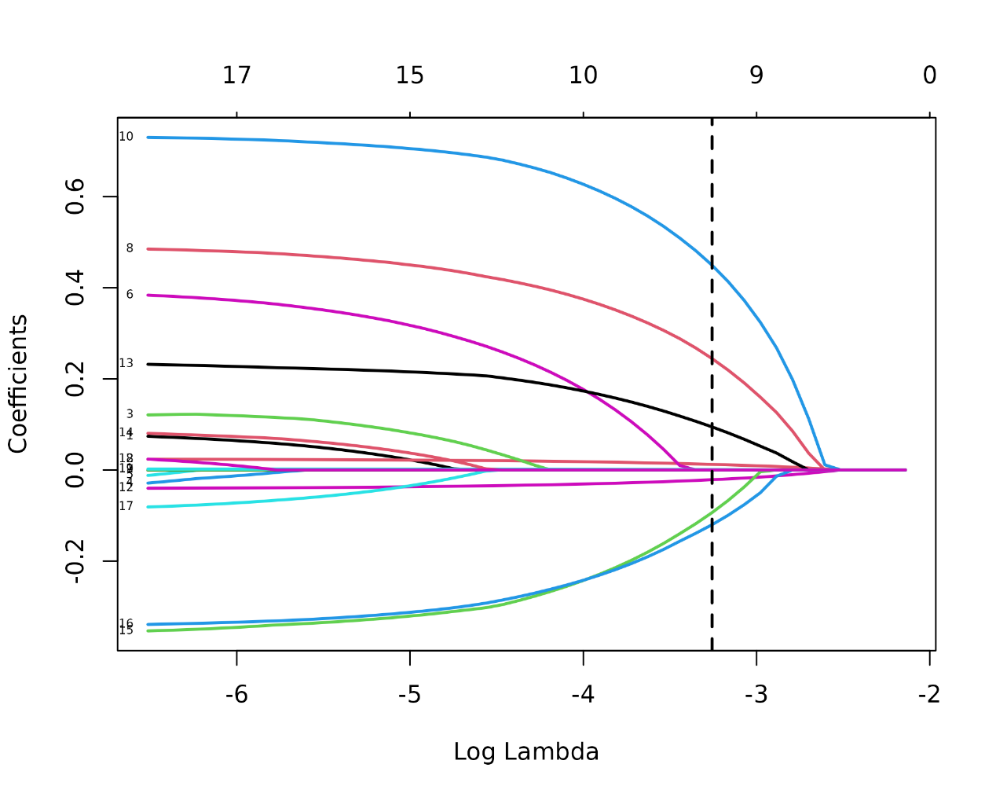


**B**

A. Optimal parameter (lambda) selection in the LASSO model used tenfold cross-validation according to the minimum standard error and lambda.1se. The partial likelihood deviance (binomial deviance) curve was plotted versus log (lambda).

B. The LASSO coefficient profiles for the 16 features were examined. A coefficient profile plot was generated using a log(lambda) sequence.
